# Mitochondrial Uptake of Thiamin Pyrophosphate: Physiological and Cell Biological Aspects

**DOI:** 10.1371/journal.pone.0073503

**Published:** 2013-08-30

**Authors:** Veedamali S. Subramanian, Svetlana M. Nabokina, Yaping Lin-Moshier, Jonathan S. Marchant, Hamid M. Said

**Affiliations:** 1 Departments of Medicine, Physiology and Biophysics, University of California Irvine, Irvine, California, United States of America; 2 Department of Veterans Affairs Medical Center, Long Beach, California, United States of America; 3 Department of Pharmacology, University of Minnesota Medical School, Minneapolis, Minnesota, United States of America; University of Florida, United States of America

## Abstract

Mammalian cells obtain vitamin B1 (thiamin) from their surrounding environment and convert it to thiamin pyrophosphate (TPP) in the cytoplasm. Most of TPP is then transported into the mitochondria via a carrier-mediated process that involves the mitochondrial thiamin pyrophosphate transporter (MTPPT). Knowledge about the physiological parameters of the MTPP-mediated uptake process, MTPPT targeting and the impact of clinical mutations in MTPPT in patients with Amish lethal microcephaly and neuropathy and bilateral striatal necrosis are not fully elucidated, and thus, were addressed in this study using custom-made ^3^H-TPP as a substrate and mitochondria isolated from mouse liver and human-derived liver HepG2 cells. Results showed ^3^H-TPP uptake by mouse liver mitochondria to be pH-independent, saturable (K_m = _6.79±0.53 µM), and specific for TPP. MTPPT protein was expressed in mouse liver and HepG2 cells, and confocal images showed a human (h)MTPPT-GFP construct to be targeted to mitochondria of HepG2 cells. A serial truncation analysis revealed that all three modules of hMTPPT protein cooperated (although at different levels of efficiency) in mitochondrial targeting rather than acting autonomously as independent targeting module. Finally, the hMTPPT clinical mutants (G125S and G177A) showed proper mitochondrial targeting but displayed significant inhibition in ^3^H-TPP uptake and a decrease in level of expression of the MTPPT protein. These findings advance our knowledge of the physiology and cell biology of the mitochondrial TPP uptake process. The results also show that clinical mutations in the hMTPPT system impair its functionality via affecting its level of expression with no effect on its targeting to mitochondria.

## Introduction

Thiamin is indispensable for normal cellular functions due to its involvement as a co-factor (mainly in the form of thiamin pyrophosphate, TPP) in critical metabolic reactions related to oxidative energy metabolism, ATP production, and reduction of cellular oxidative stress [1]–[6]; the vitamin also plays an important role in maintaining normal function/structure of the mitochondria [7]. Mammalian cells cannot synthesize thiamin endogenously, but obtain the vitamin from circulation via transport across the cell membrane; the latter process is mediated by the cell membrane thiamin transporters THTR-1 and THTR-2 (products of *SLC19A2* and *SLC19A3* genes, respectively) [8]–[10]. Following internalization of free thiamin, the majority (85 to 90%) of the vitamin is converted to thiamin pyrophosphate (TPP) via an enzymatic process that takes place exclusively in the cytoplasm [5], [11], [12]. Most of the generated TPP (∼90%) is then transported (compartmentalized) into mitochondria [13]–[15] for utilization in a variety of metabolic reactions (there is no TPP synthesis in the mitochondria; [16]). Uptake of TPP by mitochondria occurs via a carrier-mediated mechanism that involves the mitochondrial thiamin pyrophosphate transporter (MTPPT; product of the *SLC25A19* gene [17]). Previous studies that attempted at determining the physiological parameters/characteristics of the mitochondrial TPP (MTPP) uptake process have utilized either indirect enzymatic method to measure the level of the transported substrate (which as acknowledged by the authors is of limited accuracy [16]), or a yeast complementation (growth) method [18] that also does not allow accurate determination of transport kinetic parameters/characteristics.

The MTPPT (320 amino acid residues) is a member of the mitochondrial carrier family (MCF) of transporters for which a large volume of targeting data has been collected. The MCF family members exhibit a three-fold tandem repeated domain (∼100 amino acids in length), with each domain consisting of two transmembrane helices linked by a loop that contains part of the ‘signature sequence motif’ characteristic of this family of transporters (Px[D/E]xx[K/R]x[K/R]…20/30 residues.[D/E]Gxxxx[W/Y/F][K/R]G) [19], [20]. The proline residue of the signature motif enforces a sharp kink in the odd numbered helices (H1, H3 & H5) and the charged residues contribute to a salt-bridge network critical for the transport mechanism of the carrier cycle [19], [20]. Prior experiments have established that members of the MCF are synthesized without a cleavable pre-sequence but rather contain internal signaling sequences within the three modules that contribute toward targeting to mitochondria [21]–[26]. However, nothing is known specifically about the mitochondrial targeting determinants of the hMTPPT transporter and whether the different modules contribute with equal efficiency to the targeting process. Thus, we addressed these issues in the current investigation using a series of hMTPPT truncated mutants.

The *SLC25A19* gene is clinically important as mutations in this gene cause Amish congenital lethal microcephaly (an autosomal recessive disorder associated with retardation in brain development) [17], [27], [28], and neuropathy and bilateral striatal necrosis [18]. Such mutations lead to drastic depletion in mitochondria TPP level [17], [18], [27], [28]. Little, however, is known about how such clinical mutations affect the physiology and cell biology of the MTPPT system. Our aims in this study were to establish the physiological parameters and characteristics of the MTPP uptake process using a direct and accurate radiolabeled tracer method for assessing transport of very low abundant substrates, as well as determine how the MTPPT is targeted to the mitochondria. We also aimed at determining how clinical mutations in the MTPPT found in patients with Amish lethal microcephaly and neuropathy and bilateral striatal necrosis impact the function and cell biology of the MTPPT system. Thus, we used ^3^H-TPP as the substrate and mitochondria isolated from mouse liver and from human-derived liver HepG2 cells as models in our investigations. The results established the kinetic parameters of the MTPP uptake process and showed uptake is pH-independent and specific for TPP. Truncation analysis showed that all three modules of hMTPPT contribute in targeting the transporter to the mitochondria, and that the two clinical missense mutations in the hMTPPT system impair functionality of the system most likely via reduction in transport protein level of expression.

## Materials and Methods

### Materials

A GFP vector, DsRed-Mito fluorescent protein plasmid and GFP polyclonal antibodies were from Clontech (Mountain view, CA). HepG2 cells were purchased from ATCC (Manassas, VA). Normal human liver total RNA was obtained from BioChain (Nework, CA). DNA oligonucleotides were from Sigma Genosys (Woodlands, TX). ^3^H-thiamin pyrophosphate (specific activity ∼1.3Ci/mmol) was from Moravek Biochemicals (Brea, CA).

### Isolation of Mitochondria and Uptake Assays

Mitochondria were isolated from adult (∼ 3 months old) mouse liver or from HepG2 cells (maintained in Dulbecco’s modified Eagle’s medium supplemented with 10% (v/v) fetal bovine serum and 1x appropriate antibiotics), as described previously [29], [30]. Use of animals was approved by the Institutional Animal Care Use Committee (IACUC) of Veterans Affairs Medical Center at Long Beach, CA. The freshly prepared mitochondria were suspended in buffer [in mM] 140 KCl, 0.3 EDTA, 5 MgCl_2_, 10 MES, and 10 HEPES, pH 7.4, to achieve a protein concentration of ∼15–20 µg/µl and then immediately used for uptake studies using a rapid-filtration technique [31]. Briefly, mitochondria (20 µl) were added to the uptake buffer (80 µl) containing ^3^H-TPP (0.38 µM) and then subjected to the incubation at 37°C [uptake buffer was the same as suspension buffer but contained 10 mM succinate (to maintain the function of mitochondria)]. The uptake reaction was terminated after 2 min (unless otherwise stated) by the addition of 1 ml of ice-cold stop solution [in mM: 100 KCl, 100 mannitol, and 10 KH_2_PO_4_, pH 7.4] followed by rapid filtration. The filter was washed two times with stop solution followed by measurement of radioactivity in a liquid scintillation counter.

### Western Blot Analysis

Mitochondria isolated from mouse liver or HepG2 cells (∼100 µg) were subjected to protein resolution in NuPAGE 4–12% Bis-Tris gradient minigels (Invitrogen) followed by transfer onto immobilon polyvinylidene difluoride membrane (Fisher Scientific), and subsequent western blot analysis using specific antibodies. The primary antibody was rabbit polyclonal anti-MTPPT antibody (Abgent, CA) at 1 µg/ml dilution. The secondary antibody was anti-rabbit IRDye-800 antibody (LI-COR Bioscience, Lincoln, NE) at 1∶30,000 dilutions. Immunoreactive bands were visualized using the Odyssey infrared imaging system (LI-COR Bioscience) [32].

Mitochondria isolated from hMTPPT-GFP, G125S and G177A stably expressing HepG2 cells (60 µg of protein) were resolved as described above. Membranes were then incubated with anti-GFP (Clontech, CA) and anti-pyruvate dehydrogenase (PDH) antibodies (Abcam, Cambridge, MA). Immunodetection was performed by incubating the membrane with secondary antibodies [IRDye 800 labeled anti-mouse or IRDye 680 labeled goat anti-rabbit (1∶30000 dilutions for both secondary antibodies), LI-COR] for 45 min at RT. Signals were detected as described above.

### Real-Time PCR Analysis

Total RNA isolated from the mouse liver, human liver and HepG2 cells using TRIzol reagent was treated with DNase I and subjected to reverse transcription using iScript cDNA synthesis kit (Bio-Rad, Hercules, CA). The mRNA expression level was quantified in a CFX96 real-time PCR system (Bio-Rad), using iQ SYBR Green Super mix (Bio-Rad) and primers specific for mouse MTPPT (forward: 5′-TCCAGATTGAACGCCTGTG -3′ and reverse: 5′-GACAGCTCCGTAGCCTATGGAC -3′) and acidic ribosomal phosphoprotein (ARPO) (forward: 5′- GCTGAACATCTCCCCCTTCTC-3′ and reverse: 5′-ATATCCTCATCTGATTCCTCC-3′). The hMTPPT (forward: 5′-AGCATGAGCGCCTGTCGC-3′ and reverse: 5′-TGAGCTGGGACHTGTCCTTTCCA-3′) and human β-actin (forward: 5′-AGCCAGACCGTCTCCTTGTA-3′ and reverse: 5′- TAGAGAGGGCCCACCACAC-3′). Real-time PCR conditions were used as previously described [32]. Data were normalized to ARPO or β-actin and calculated using a relative relationship method supplied by the iCycler manufacturer (Bio-Rad) [33].

### Generation of hMTPPT Full-length, Truncated and Mutated Constructs

The full-length GFP-hMTPPT and hMTPPT-GFP, and truncated constructs were generated by PCR amplification using the hMTPPT specific primer combinations (Table 1) and conditions as described previously [34], [35]. The GFP vectors and PCR products were digested with *Hind III* and *Sac II,* and the respective products were gel separated and ligated to generate in-frame fusion constructs with the GFP fluorescent protein fused to the NH_2_/COOH-terminus of the construct. The Quick change™ site-directed mutagenesis kit (Stratagene, CA) was used to introduce insertions or deletions of nucleotides into the open reading frame (ORF) of *hMTPPT (SLC25A19)*. Overlapping primers containing the mutated nucleotides to the specified mutation sites (Table 1), and full-length hMTPPT-GFP fused plasmid was used as a template for PCR based site-directed mutagenesis as described before [35]. The nucleotide sequences of full-length, truncated and mutated constructs were verified by sequencing (Laragen, Los Angeles, CA).

**Table 1:** Combination of primers used to prepare the full length, truncated and mutated constructs of hMTPPT by PCR.

Construct	Forward and Reverse Primers (5′-3′)	Positions (bp)	Fragment (bp)
hMTPPT[1–320]-GFP/GFP-hMTPPT[1–320]	CCC**AAGCTT**ATGGTTGGCTATGACCCC; TCC*CCGCGG*GCGCTGGCTGGCTGTCCT	1–960	960
hMTPPT[1–63]-GFP	CCC**AAGCTT**ATGGTTGGCTATGACCCC; TCC*CCGCGG*GAGGATGCCATGGTACTT	1–189	189
hMTPPT[1–164]-GFP	CCC**AAGCTT**ATGGTTGGCTATGACCCC; TCC*CCGCGG*GGTCCCCACGGCGTGGCG	1–492	492
hMTPPT[64–320]-GFP	CCCAAGCTTATGCAGGCCTCTAGGCAGATT; TCC*CCGCGG*GCGCTGGCTGGCTGTCCT	190–960	770
hMTPPT[1–265]-GFP	CCC**AAGCTT**ATGGTTGGCTATGACCCC; TCC*CCGCGG*GAGGCCCTTGTATCTCCG	1–795	895
hMTPPT[266–320]-GFP	CCCAAGCTTATGGACTGTGCCAAGCAG; TCC*CCGCGG*GCGCTGGCTGGCTGTCCT	796–960	164
hMTPPT[64–265]-GFP	CCCAAGCTTATGCAGGCCTCTAGGCAGATT; TCC*CCGCGG*GAGGCCCTTGTATCTCCG	190–795	605
hMTPPT[165–320]-GFP	CCC**AAGCTT**ATGTATAGGAGCGAAGGC; TCC*CCGCGG*GCGCTGGCTGGCTGTCCT	493–960	467
hMTPPT[102–207]-GFP	CCC**AAGCTT**ATGCTGACGGAGCTGGTC; TCC*CCGCGG*TATGGCCCACTTGTACAG	304–622	318
hMTPPT[102–320]-GFP	CCC**AAGCTT**ATGCTGACGGAGCTGGTC; TCC*CCGCGG*GCGCTGGCTGGCTGTCCT	304–960	656
hMTPPT[G125S]-GFP	CACTTTGTATGT***AGT***GGCCTGGCTGCC; GGCAGCCAGGCC***ACT***ACATACAAAGTG		
hMTPPT[G177A]-GFP	GTTTTCTACAAA***GCC***TTGGCTCCCACC GGTGGGAGCCAA***GGC***TTTGTAGAAAAC		

Restriction sites *Hind III* (boldface text) and *Sac II* (italic text) were added to the hMTPPT primers to allow subsequent sub-cloning into the GFP vectors and mutant primers are in bold italic text.

### Transient and Stable Transfections

For transient transfection, HepG2 cells were grown on sterile glass-bottomed Petri dishes (MatTek Corporation, Ashland, MA) and transfected at 90% confluency (4 µg plasmid DNA) using 4 µl of Lipofectamine2000 (Invitrogen, CA). After 24–48 hrs, cells were analyzed by confocal microscopy. For stable transfection, HepG2 cells were selected using G418 (0.5 mg/ml) for 6–8 weeks.

### Xenopus Oocyte Preparation and Nuclear Microinjection

Adult female *Xenopus laevis* frogs were anesthetized by immersion in 0.1% aqueous solution of 3-aminobenzoic acid ethyl ester (MS-222) for 15 min, and after death by decapitation, whole ovaries were removed following procedures approved by University of Minnesota IACUC. The epithelial layers of stage VI oocytes were removed and treated with collagenase (0.5 mg/ml for 30 min) in dissociation solution (in mM: 82.5 NaCl, 2.5 KCl, 10 Na_2_HPO_4_, and 5 HEPES, pH 7.8) to ensure complete defolliculation. For expression studies, ∼2 ng of plasmid cDNA in 5 nl of intracellular solution (in mM: 140 KCl, 10 HEPES, 3 MgCl_2_, 1 EGTA, and 0.5 CaCl_2_, pH 7.4) were injected using established methods [36], [37]. Injected oocytes were separated and maintained in Barth’s solution for 24–48 hrs. Western blotting of *Xenopus* oocytes was performed as described [38].

### Confocal Imaging of hMTPPT Full-length, Truncated and Mutant Constructs

HepG2 cell monolayers grown on cover-slip petri-dishes were imaged using a Nikon C-1 confocal scanner head attached to a Nikon Inverted phase contrast microscope. The fluorophores were excited using the 488-nm (GFP) or 543-nm laser lines and emitted fluorescence was monitored with a 515±30 nm band pass (GFP) or at 620±60 nm long-pass (Red) filter as described before [34], [35].

### Data Presentation and Statistical Analysis

Data are presented as mean ± SE of multiple uptake determinations and expressed in picomoles or femtomoles per milligram of protein per unit of time. Student’s *t*-test was used in statistical analysis with statistical significance set at p<0.05. Kinetic parameters of the saturable TPP uptake process [(i.e., maximal velocity (V_max_) and the apparent Michaelis-Menten constant (K_m_)] were calculated [39]. Uptake by the saturable component at certain TPP concentration was calculated by subtracting uptake by simple diffusion which was determined from the slope of the line between the point of origin and uptake at high pharmacological concentration of TPP (1 mM) from total TPP uptake.

## Results and Discussion

### Characteristics and Kinetics of ^3^H-TPP Uptake by Freshly Isolated Mitochondria

**Figure 1:**
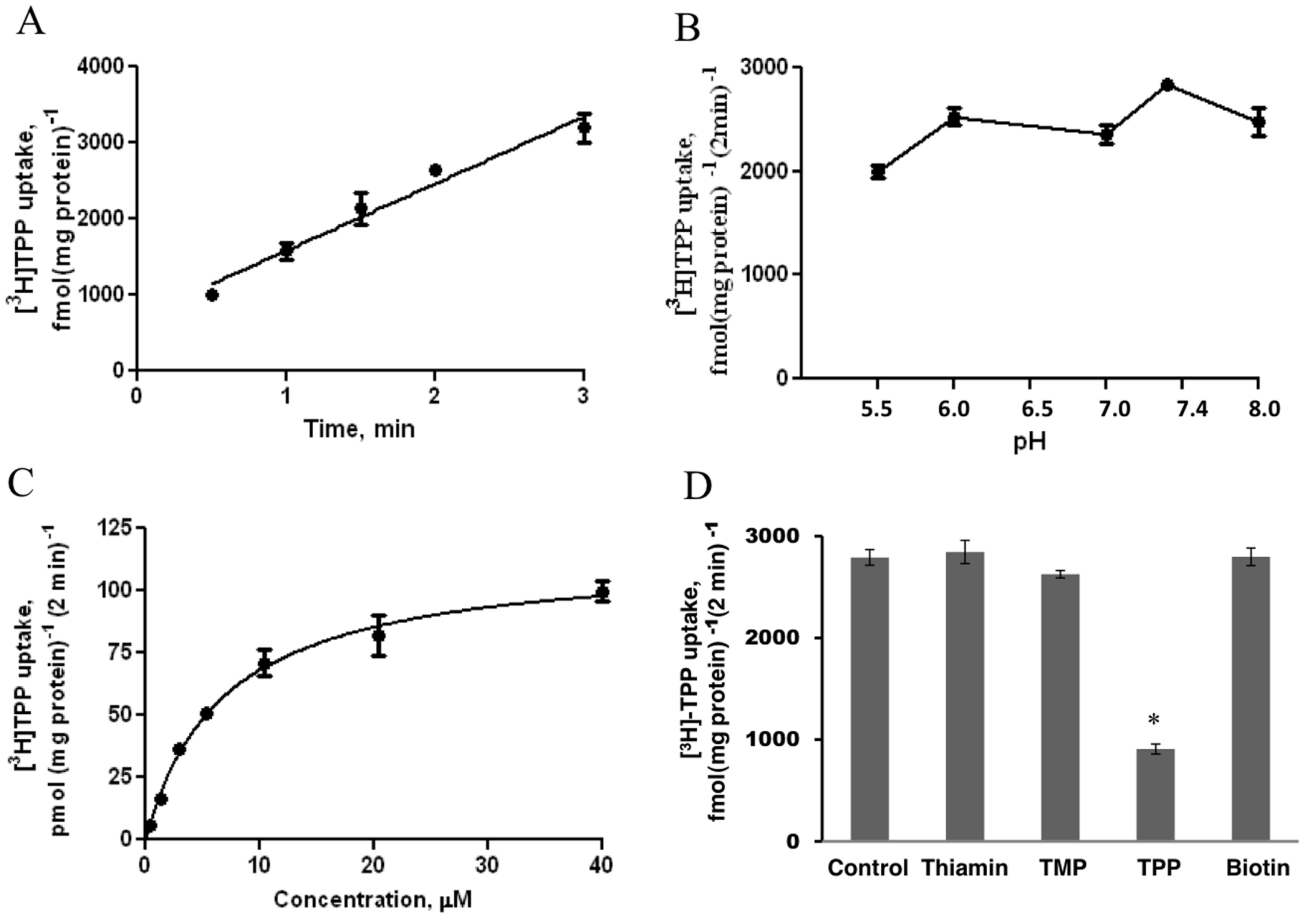
Characteristics of the TPP uptake process by the isolated mouse liver mitochondria. **A)** Uptake as a function of time. Mitochondria were incubated in uptake buffer, pH 7.4, at 37°C. ^3^H-TPP (0.38 µM) was added to the incubation medium at the start of uptake. Data are mean ± SE of 3–4 separate uptake determinations. **B)** Effect of incubation buffer pH on the initial-rate of ^3^H -TPP uptake. Mitochondria were incubated in uptake buffer of varying pH at 37°C. ^3^H-TPP (0.38 µM) was added to the incubation buffer at the onset of a 2 min of incubation time (i.e., initial-rate). Data are mean ± SE of 3–4 separate uptake determinations. **C)** Uptake of ^3^H-TPP as a function of substrate concentration. Mitochondria were incubated in uptake buffer, pH 7.4, at 37°C in the presence of different concentrations of TPP. Uptake was measured after 2 min incubation (i.e., initial-rate). Uptake by the carrier-mediated system was calculated as described in “Methods”. Data are mean ± SE of 3–4 separate uptake determinations. **D)** Effect of structural analogs of TPP on the initial-rate of uptake. Mitochondria were incubated in uptake buffer, pH 7.4, at 37°C in the presence of ^3^H-TPP (0.38 µM) and unlabeled TPP, TMP, thiamin, and biotin (120 µM for all). Uptake was measured after 2 min incubation (i.e., initial-rate). Data are mean ± SE of 5–12 separate uptake determinations. *p<0.01.

Figure 1A shows the results of uptake of low physiological concentration of ^3^H-TPP (0.38 µM) by mouse liver mitochondria as a function of time. Uptake was found to be linear [r = 0.97] for up to 3 min of incubation (pH 7.4) and occurred at a rate of 881.0±87.71 fmol (mg protein)^−1^. Similarly, uptake of high concentration of TPP (40 µM) was linear for up to 3 min of incubation (data not shown). We chose a 2 min incubation time to represent the initial-rate of uptake in all our subsequent studies. Figure 1B depicts the results on the effect of buffer pH on TPP (0.38 µM) uptake by mouse liver mitochondria over the range of pH 5.5–8.0. Over the examined range, the rate of TPP uptake fluctuated slightly without clear preference to acidic, neutral or alkaline pH. Considering this pH-independent nature, we performed subsequent studies at pH 7.4. Next, we examined the uptake of TPP as a function of substrate concentration (0.38–40 µM TPP) in order to determine the kinetic parameters of the uptake process. The results (Figure 1C) showed the initial-rate of uptake of ^3^H-TPP by mitochondria to be saturable. Kinetic parameters of the saturable component were determined as described in “Methods” and found to be of 6.79±0.53 µM for the apparent K_m_, and 114.3±3.08 pmol (mg protein)^−1^ (2 min)^−1^ for the V_max_.

To determine specificity of the mitochondrial uptake process, we examined the effect of unlabeled TPP, its analogs/derivatives including free thiamin and thiamin monophosphate (TMP), as well as the structurally unrelated vitamin biotin (all at 120 µM) on the initial rate of ^3^H-TPP (0.38 µM) uptake. The results showed that with the exception of unlabeled TPP, which caused a significantly (p<0.01) inhibition in ^3^H-TPP uptake, none of the other compounds affected ^3^H-TPP uptake (Figure 1D).

### Expression of MTPPT (SLC25A19) in Mouse and Human Liver, and HepG2 Cells

**Figure 2:**
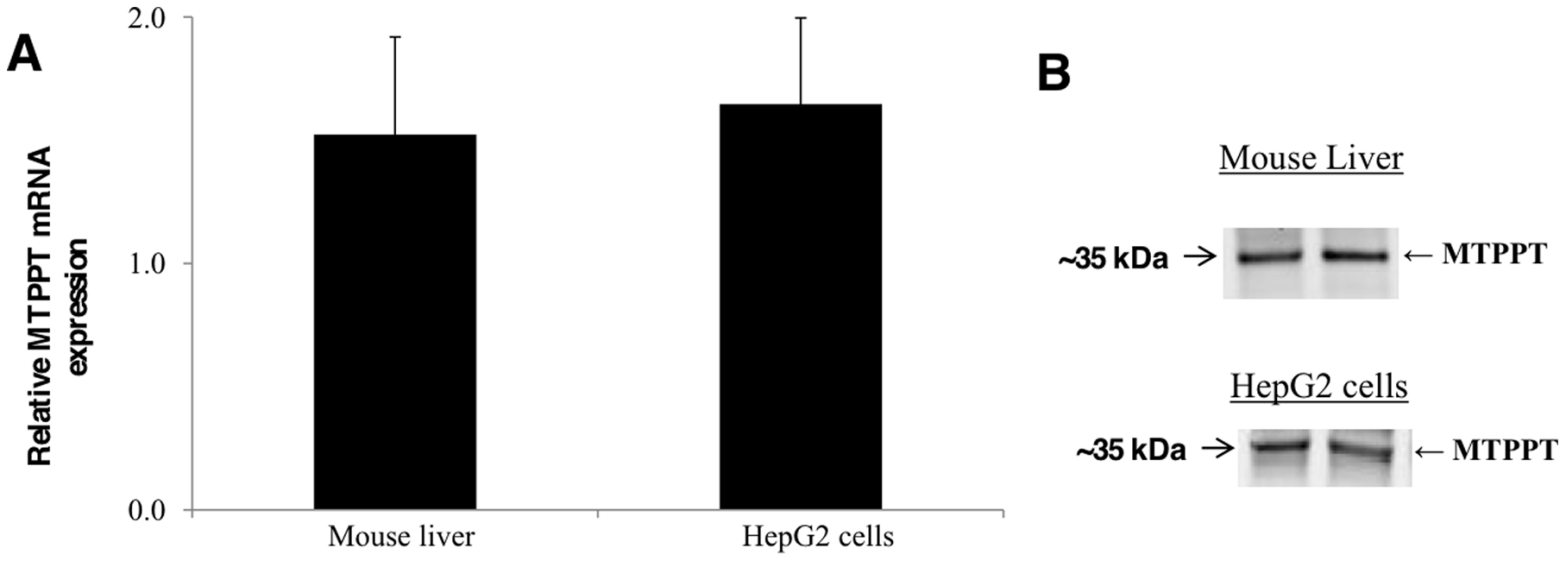
Expression of MTPPT in the mouse liver and HepG2 cells at the mRNA and protein levels. **A)** Total RNA was isolated and quantitative real-time PCR was performed as described in “Methods”. Data (mean ± SE) presented are from four independent mice normalized relative to ARPO or multiple samples from HepG2 cells were normalized relative to β-actin. **B)** Western blot analysis was performed with the use of isolated mouse liver or HepG2 cells mitochondria (100 µg) and specific anti-MTPPT polyclonal antibodies as described in “Methods”. Data from a representative experiment involving two separate mitochondria preparations from two mice or HepG2 cells are shown.

The presence of MTPPT mRNA (product of *SLC25A19* gene) was studied in mouse liver and in human-derived liver HepG2 cells by mean of RT-PCR using specific primers and expression was resolved in each of these preparations (Figure 2A), similar expression of the MTPPT mRNA was observed in human liver (data not shown). We also observed expression of the MTPPT protein in mouse liver and HepG2 cells studies using Western blotting with anti-MTPPT polyclonal antibodies (Figure 2B).

### Targeting of the hMTPPT to Mitochondria

**Figure 3:**
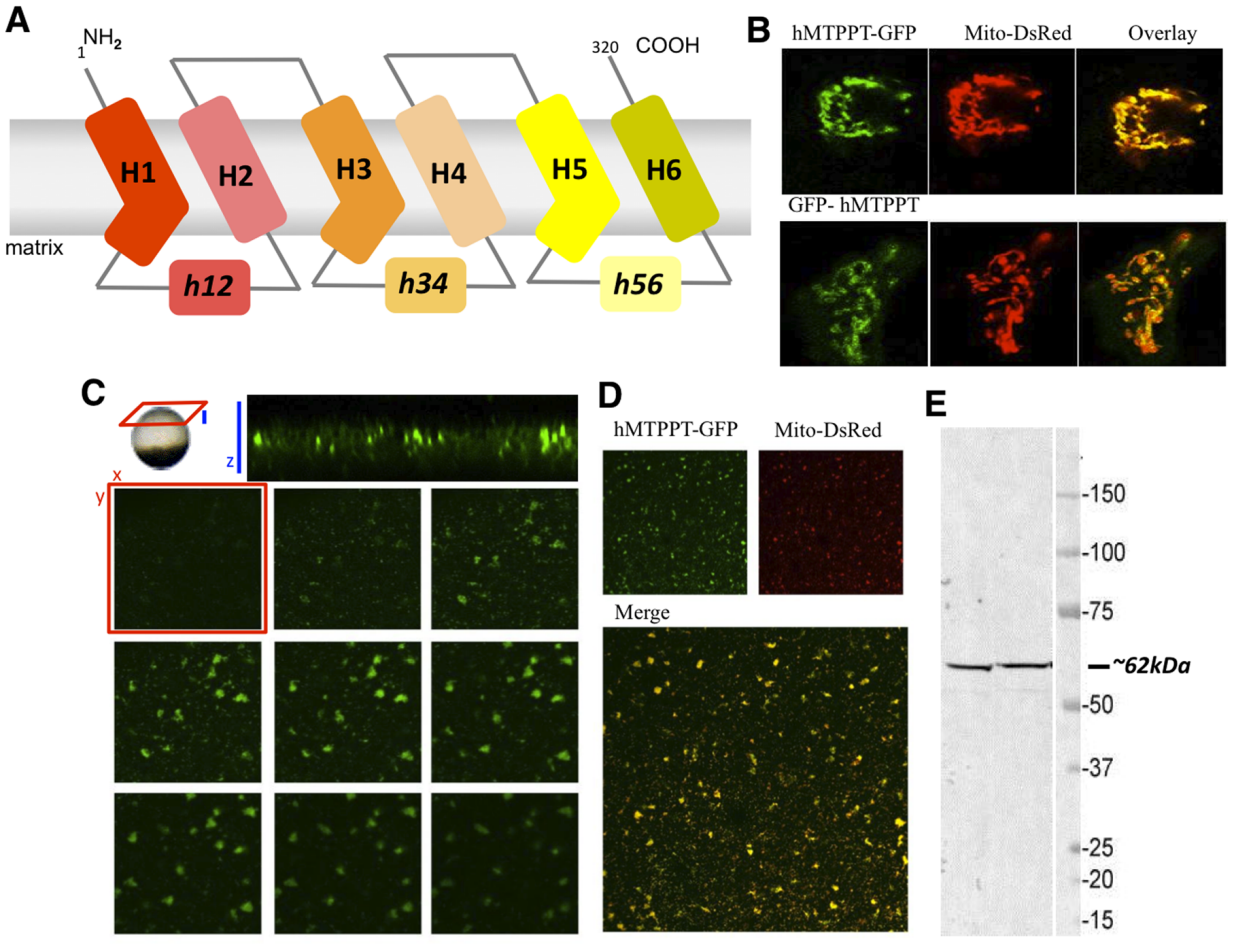
Cellular distribution of hMTPPT-GFP or GFP-hMTPPT in HepG2 cells and *X*. Oocytes. **A)** Predicted 6 transmembrane domains of hMTPPT showing the location of three-fold tandem repeated domains and both NH_2_ and COOH-terminals oriented towards the cytosol. **B)** Lateral (*xy*) confocal images of hMTPPT-GFP or GFP-hMTPPT co-transfected with DsRed-Mito (mitochondrial marker) and their overlay in HepG2 cells. **C)** Resolution of hMTPPT-GFP expression in a *Xenopus* oocyte. Top, axial image (‘xz’, blue) of a cortical band of hMTPPT-GFP expression. Individual lateral (‘xy’, red) images are displayed as image planes taken at successive 1 µm intervals into the oocyte cortex. **D)** Lateral (*xy*) confocal images of hMTPPT-GFP co-transfected with DsRed-Mito and their overlay in *Xenopus* oocyte. **E)** Western blot was performed on two individual hMTPPT-GFP expressing oocytes and probed with mouse anti-GFP (1∶1000) and goat anti-mouse antibodies (1∶5000).

After initial characterization of the ^3^H-TPP transport process into native mouse liver mitochondria, we investigated the determinants that dictate the targeting of MTPPT to mitochondria using cultured HepG2 cells. This system was chosen owing to experimental tractability overcoming the limitations in transfection and imaging in native mouse liver. hMTPPT is predicted to harbor 6 transmembrane domains with NH_2_ and COOH terminals oriented towards the cytosol (Figure 3A). To visualize the targeting of hMTPPT, NH_2_ and COOH terminal fusions of the full-length hMTPPT cDNA to the green fluorescent protein were constructed (GFP-hMTPPT, hMTPPT-GFP). These constructs were transiently transfected into HepG2 cells together with a mitochondrial marker (DsRed-Mito) and the resulting fluorescence distribution imaged using confocal microscopy. Lateral (xy) images of transfected HepG2 cells showed that both hMTPPT constructs targeted to the mitochondria regardless of the positioning of the fluorescent protein tag (Figure 3B). Similar mitochondrial targeting of hMTPPT-GFP cDNA was observed when the construct was heterologously expressed in *Xenopus* oocytes by nuclear microinjection (Figure 3C). hMTPPT-GFP expression was visible within a broad band of expression in the oocyte sub-cortex (Figure 3C), with lateral (‘xy’) confocal sections revealing the clumps and vermiform structures diagnostic of mitochondrial morphology in the *Xenopus* oocyte [40]. Figure 3D shows the hMTPPT-GFP and DsRed-Mito co-expressing oocytes displays strong colocalization. Single oocyte western blots revealed migration of the fluorescent protein-tagged fusion construct at ∼62 kDa, consistent with expression of a ∼35 kDa (untagged) transporter construct (compare with Figure 3E).

### Role of the Multiple Modules in Targeting of the hMTPPT to Mitochondria

**Figure 4:**
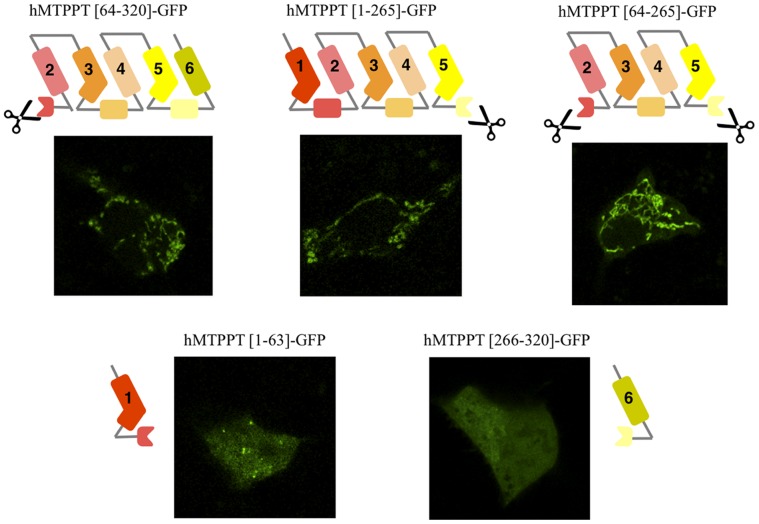
Systematic truncation of the hMTPPT polypeptide and their expression pattern in HepG2 cells. Sub cellular expression of indicated (five) hMTPPT-GFP truncated constructs in HepG2 cells. Lateral (*xy*) confocal images of different truncated constructs were imaged after 24–48 hrs of transient transfection.

**Figure 5:**
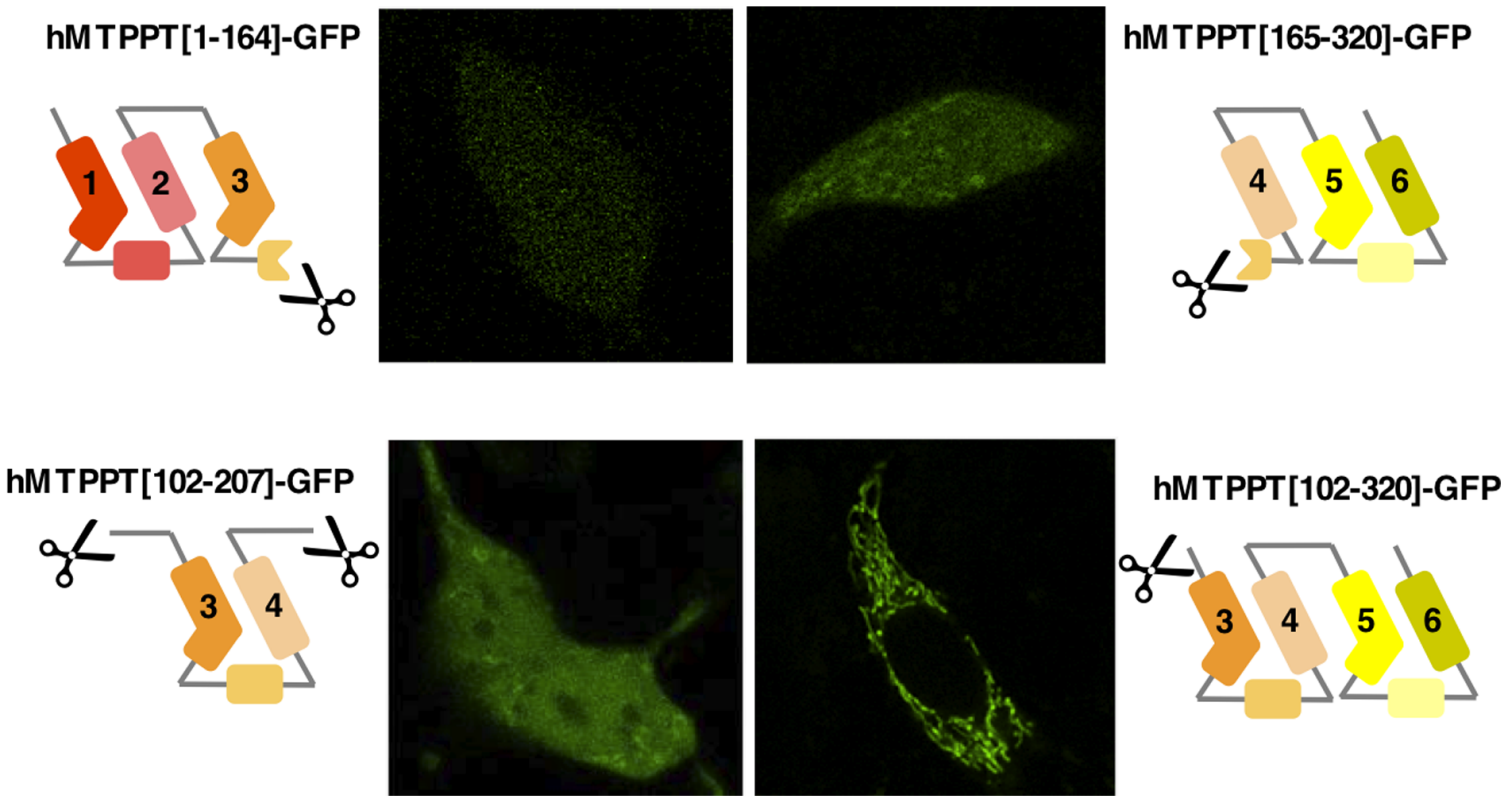
Systematic truncation of the hMTPPT polypeptide and their expression pattern in HepG2 cells. Sub cellular expression of indicated (four) hMTPPT-GFP truncated constructs in HepG2 cells. Lateral (*xy*) confocal images of different truncated constructs were imaged after 24–48 hrs of transient transfection.

To delimit regions of hMTPPT important for mitochondrial targeting a series of truncated constructs were prepared (Figures 4 & 5). On the basis of prior data implicating a key role for the signature motif in hMTPPT targeting and translocation [21], [22], we designed truncations to interrupt the signature motif in each hMTPPT module, i.e. truncation between helices H1 and H2, H3 and H4, H5 and H6. The results of these manipulations were as follows.

First, disruption of the motif between H1 and H2 failed to impair mitochondrial targeting of the residual construct (hMTPPT[64–320]-GFP) (Figure 4). Truncation between H5 and H6, which disrupted the third signature motif, also did not impact the targeting of the parent construct (hMTPPT[1–265]-GFP, Figure 4) which co-localized with DsRed-Mito (data not shown) (Figure 4). The residual truncated sequences (hMTPPT[1–63]-GFP and hMTPPT[266–320]-GFP) when fused to GFP resulted in predominantly cytosolic fluorescence (Figure 4). Therefore, the integrity of the first or the third signature motif proved non-essential for mitochondrial targeting of hMTPPT. This conclusion was underscored by the observation that the double truncated mutant hMTPPT[64–265]-GFP also localized to mitochondria (Figure 4). This construct comprised solely the second module of hMTPPT (H3–H4) flanked by an additional transmembrane helix at the NH_2_ (H2) and COOH terminus (H5).

Next, hMTPPT was cleaved within the second signature motif (i.e. in half) resulting in two constructs (hMTPPT[1–164]-GFP and hMTPPT[165–320]-GFP, Figure 5). Neither construct co-localize with DsRed-Mito (data not shown) suggesting that integrity of this region of hMTPPT might be important for appropriate mitochondrial targeting. While the second module appeared necessary for mitochondrial targeting, it was not sufficient by itself as this region alone (hMTPPT[102–207]-GFP) failed to express in mitochondria. Mitochondrial targeting was shown to be enhanced by flanking transmembrane regions at both the NH_2_ and COOH terminus (hMTPPT[63–265]-GFP, see above), or NH_2_ terminus alone (hMTPPT[102–320]-GFP) (Figure 5).

These analyses reinforce the view that the modules of MCF proteins cooperate in targeting of these carriers to mitochondria [21] rather than each act autonomously as independent targeting modules. Additionally, our truncation data point to a non-equivalency in the modules, namely that the H3-(h34)-H4 region appears necessary for efficient targeting to the mitochondria. Bioinformatic analysis suggests the second module (containing the H3-(h34)-H4 region) conforms to the consensus signature motif present in modules 1 and 3 of hMTPPT, unique feature being a shorter length (40 amino acids) compared with modules 1 and 2 (47–49 residues, respectively). Our finding above showing a greater role for the second module of hMTPPT in mitochondrial targeting is in contrast to some other observations with other members of the MCF which showed prominent role for other modules in the targeting event [41].

### Effect of Clinical Mutations on Physiology/Cell Biology of hMTPPT

**Figure 6:**
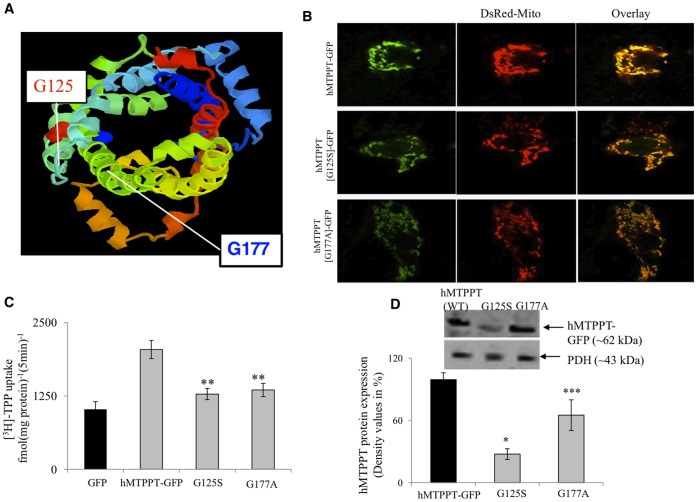
Cellular expression of hMTPPT clinical mutants and their function in stable HepG2 cells. **A**) The Raswin program which predicts the location of the clinical mutants (G125S and G177A) in the hMTPPT polypeptide. **B)** Lateral (*xy*) scan of hMTPPT-GFP (WT) and clinical mutants were co-transfected with DsRed-mito and their overlay in stable HepG2 cell line. **C**) ^3^H-TPP (0.38 µM)) uptake by stable HepG2 cells expressing GFP (vector alone), hMTPPT-GFP (WT) and clinical mutants. Initial-rate of ^3^H-TPP uptake was performed on mitochondria isolated from HepG2 cells. The uptake was linear up to 10 min (data not shown). **D**) Western blot analyses were performed on isolated mitochondria from hMTPPT (WT) and clinical mutants (60 µg protein) as described in “Methods”. Blots were incubated with mouse monoclonal GFP antibodies. Samples were normalized relative to pyruvate dehydrogenase (PDH) protein expression. Data are mean ± SE from at least three different samples from three different batches of cells. * p<0.01,** p<0.02,*** p<0.04.

Two disease-linked mutations have been identified in *SLC25A19* [17], [18], [27], [28]. First identified was a G177A (c.530G>C) substitution [17], [27], [28] associated with Amish lethal microcephaly which is an inherited autosomal recessive disorder which markedly affects brain development and leads to alpha-ketoglutaric aciduria. The mutated residue comprises the terminal glycine within the MCF signature motif found between transmembrane helices H3 and H4 (Px[D/E]xx[K/R]x[K/R].20/30.[D/E]Gxxxx[W/Y/F][K/R]G). This residue is highly conserved in MCF family members, and localizes about one and a half helix turns (between helices h_34_ and H4) below the substrate binding sites (Figure 6A). The second disease-associated mutation in *SLC25A19* is a G125S allelic variant that is associated with neuropathy and bilateral striatal necrosis [18]. This conserved glycine residue this time located at the start of H3, within the ‘P-G level 1’ region (Figure 6A).

We experimentally introduced point mutations into hMTPPT to mimic both these clinical mutations (hMTPPT[G125S]-GFP and hMTPPT[G177A]-GFP). The hMTPPT[G125S]-GFP and hMTPPT[G177A]-GFP mutants were transfected into HepG2 cells and stable cell lines prepared for each mutant. After selection, stable HepG2 cells were co-transfected with DsRed-Mito. The confocal imaging revealed that both two missense hMTPPT mutants exhibited mitochondrial expression in HepG2 cells (Figure 6B). Therefore these clinical phenotypes did not derive from mistargeting of hMTPPT.

Next, we examined the effect of the impact of both clinical mutations on the functionality of transporter, by quantifying ^3^H-TPP uptake into mitochondria in stable HepG2 cell lines. The uptake results revealed a significant inhibition in ^3^H-TPP uptake (p<0.02 for both mutants) in both hMTPPT[G125S]-GFP and hMTPPT[G177A]-GFP expressing cell lines when compared with those expressing the wild-type hMTPPT-GFP construct or GFP alone (Figure 6C). Note, this is the first study utilizing the native TPP as a substrate to examine the functionality of clinical mutations in isolated mitochondria. The observed decrease in ^3^H-TPP accumulation was consistent with proposed structural roles of the mutated residues in hMTPPT functionality. For G177A, this residue lies in close proximity to one of the salt bridges (K241 to D140) in the cytosolic conformation of the carrier (‘c-state’) that may serve to close the base of the water-filled cavity of the transporter [20]. The glycine residue has been proposed to act as a hinge residue (within P-G level 2, [19]) serving to open/close the carrier on the matrix side during the carrier transport cycle. For G125S also, this region has been implicated as a hinge region involved in conformational changes needed to translocate substrate [19]. This region is found in the three-dimensional structure about one helical turn above substrate binding region toward the cytosolic face. Consistent with prior findings (liposomes [17], [27] vertebrate [42] and yeast [43]) our data demonstrated impaired ^3^H-TPP uptake functionality when substrate transport was assessed by HepG2 cells mitochondria (Figure 6C). Determination of expression levels of the different hMTPPT (WT) and mutant constructs were then assessed by western blot analysis with anti-GFP antibodies. Densitometric analysis of several blots revealed a significant decrease in the protein expression of the clinical mutants compared with wild-type hMTPPT (p<0.01 for G125S and p<0.04 for G177A, Figure 6D). Therefore, the observed loss of function phenotype likely results from both impaired protein expression as well as impaired transport capabilities first revealed by protein reconstitution experiments [17], [27].

In summary, these studies have established the kinetic parameters and characteristics of the mitochondrial TPP uptake process using a sensitive radio-isotope approach. The results also showed that all three modules of hMTPPT protein are essential for proper mitochondrial targeting, although they participate at a variable degree of efficiency. Furthermore the results show that clinical mutations in the hMTPPT system impair its functionality via affecting the level of expression of the transport protein with no impairment in its targeting to mitochondria.
