# Correction: Mitochondrial Uptake of Thiamin Pyrophosphate: Physiological and Cell Biological Aspects

**DOI:** 10.1371/journal.pone.0186541

**Published:** 2017-10-11

**Authors:** Veedamali S. Subramanian, Svetlana M. Nabokina, Yaping Lin-Moshier, Jonathan S. Marchant, Hamid M. Said

The following information is missing from the Funding section: This study was supported by NIH grant AA 18071 to HMS.
